# Enhanced Recovery After Surgery (ERAS) Protocols and Their Impact on Quality of Care and Patient Safety in Peri-Anaesthesia Nursing: A Narrative Review

**DOI:** 10.7759/cureus.101821

**Published:** 2026-01-19

**Authors:** Maria Bourazani, Despoina Pappa, Panagiota Manthou, Maria Chrysi, Christos Fasianos

**Affiliations:** 1 Anesthesiology, Hellenic Anticancer Institute, Saint Savvas Hospital, Athens, GRC; 2 Nursing, Henry Dunant Hospital Center, Athens, GRC; 3 Infection Prevention, University of West Attica, Athens, GRC; 4 Surgery, Hellenic Anticancer Institute, Saint Savvas Hospital, Athens, GRC; 5 Health, Hellenic Anticancer Institute, Saint Savvas Hospital, Athens, GRC

**Keywords:** enhanced recovery after surgery, eras protocols, patient safety, peri-anesthesia nursing, perioperative care, quality improvement, quality of care

## Abstract

This narrative review aimed to critically synthesise current evidence on Enhanced Recovery After Surgery (ERAS) protocols and their impact on quality of care and patient safety in peri-anaesthesia nursing, with particular emphasis on the Post-Anaesthesia Care Unit (PACU) and the immediate postoperative period. A narrative literature review was conducted to identify evidence related to ERAS implementation and peri-anaesthesia nursing practice. International databases, including PubMed and Elsevier, were searched for relevant publications addressing ERAS protocols, perioperative care, patient safety, and quality improvement. The review focused on studies published between 2019 and 2025. Peer-reviewed articles, clinical guidelines, and original studies relevant to peri-anaesthesia nursing and postoperative recovery were included, and the selected literature was screened and synthesised descriptively.

The review demonstrated that ERAS protocols are associated with improved quality and patient safety outcomes during the peri-anaesthesia phase of care. Reported benefits included enhanced postoperative recovery and pain management, reduced complication rates, earlier mobilisation, and greater consistency in clinical practice within the PACU. The findings also highlighted the central role of peri-anaesthesia nurses in patient monitoring, education, and early recovery support, underscoring their contribution to successful ERAS implementation. Overall, ERAS protocols provide a structured framework for improving quality and patient safety in peri-anaesthesia nursing care. Strong peri-anaesthesia nursing involvement and effective multidisciplinary collaboration are essential for optimal implementation, particularly during immediate postoperative recovery. Further research focusing on peri-anaesthesia nursing-specific outcomes and experiences with ERAS pathways is warranted.

## Introduction and background

Perioperative care of surgical patients is characterised by increased complexity and the involvement of multiple healthcare professionals, rendering the delivery of safe, high-quality care a demanding process. Surgical procedures are associated with an increased risk of complications, prolonged hospitalisation, and deterioration of patients’ quality of life, highlighting the need for structured, evidence-based approaches to care [1].

Enhanced Recovery After Surgery (ERAS) protocols were developed in response to these challenges, with the aim of optimising perioperative care through the systematic application of evidence-based interventions [2]. Over recent decades, ERAS pathways have been implemented across a wide range of surgical specialities, including general surgery, orthopaedics, urology, and gynaecology [3-7]. Their growing adoption reflects a paradigm shift from traditional perioperative practices towards standardised, patient-centred, and outcome-oriented models of care [8].

The peri-anaesthesia phase, including immediate postoperative recovery and care in the Post-anaesthesia Care Unit (PACU), represents a high-risk period for complications such as pain, nausea and vomiting, respiratory events, and delayed mobilisation. ERAS protocols provide structured guidance that supports peri-anaesthesia nurses in monitoring, symptom management, patient education, and recovery optimisation during this critical phase.

Within this context, ERAS protocols should not be viewed solely as clinical tools but also as instruments for improving quality and patient safety in perioperative care, supporting consistency in practice and fostering multidisciplinary teamwork [9].

The aim of this study was to review and critically examine ERAS protocols as a tool for improving quality and patient safety in perioperative care, with particular emphasis on peri-anaesthesia nursing practice, standardisation of clinical care, patient safety, and the role of nursing during the immediate postoperative and recovery phases.

## Review

Research methodology

A narrative literature review was conducted covering the period from 2019 to 2025. A literature search of international electronic databases was performed, including PubMed and Elsevier. Keywords were used in the English language. The initial search included terms and combinations such as “Enhanced Recovery After Surgery”, “ERAS protocols”, “perioperative care”, “patient safety”, “quality of care” and “quality improvement”. Relevant articles published in peer-reviewed journals were screened based on title and abstract, and those addressing ERAS implementation, quality, and patient safety in perioperative care were included.

Inclusion criteria encompassed studies published between 2019 and 2025, written in the English language and available in free full text. Eligible studies examined the application of ERAS protocols within perioperative care and reported outcomes related to quality of care, patient safety, standardisation of clinical practice, and/or the role of nursing care. Both original research studies and review articles published in peer-reviewed journals were included. The methodological quality and potential risk of bias of the included studies were assessed descriptively, taking into account study design, clarity of methodological reporting, and consistency of outcome measures. Due to heterogeneity across study designs and outcomes, risk of bias was considered in the interpretation of findings rather than through a formal quantitative assessment.

Results

**Figure 1:**
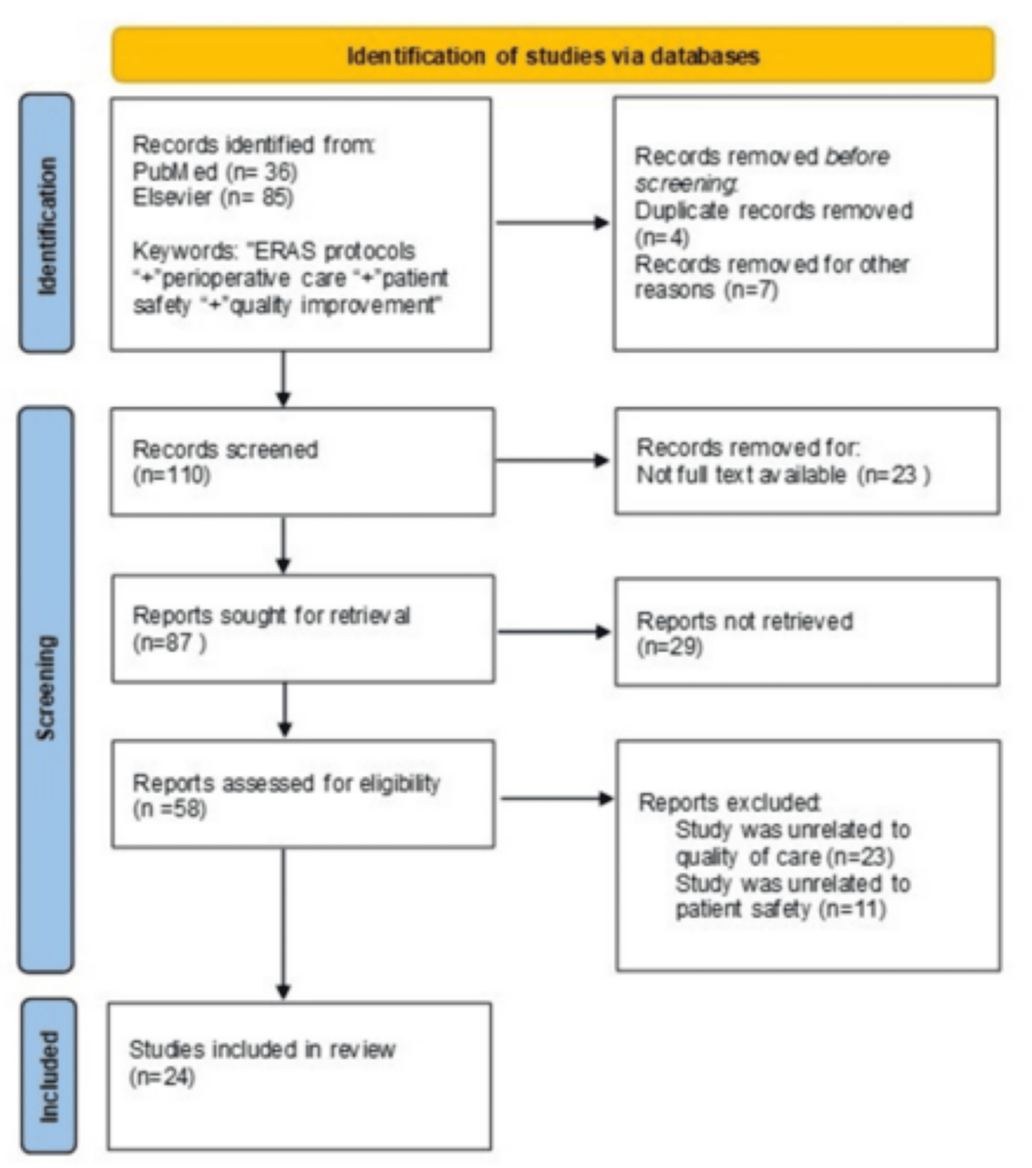
Flowchart of the literature review process

Based on the inclusion criteria, a total of 24 publications were reviewed. Of these, most studies investigated the implementation of ERAS protocols across different surgical specialities and their effects on perioperative outcomes, quality of care, and patient safety. Several publications highlighted the role of standardised clinical pathways in reducing variability in practice, while others emphasised the importance of nursing involvement and multidisciplinary collaboration in ERAS implementation. Overall, the findings consistently demonstrated that ERAS protocols are associated with improved recovery, reduced complications, and enhanced quality and safety in perioperative care. Figure 1 illustrates the flowchart of the literature review process. 

Table 1 lists the author names, reference numbers, and major findings for each study.

**Table 1:** Key studies related to ERAS, quality improvement, and patient safety ERAS, Enhanced Recovery After Surgery

Author	Ref.	Major findings
Ljungqvist and Hubner	[1]	Describes ERAS principles and demonstrates that ERAS protocols are feasible and beneficial in elderly patients, with improved recovery and reduced complications when appropriately adapted.
Gustafsson et al.	[2]	Provides comprehensive ERAS Society guidelines for elective colorectal surgery, showing strong evidence for reduced postoperative complications, shorter length of stay, and improved outcomes.
Nelson et al.	[3]	Reports successful large-scale implementation of multiple ERAS guidelines in Alberta, Canada, associated with improved compliance and better postoperative outcomes.
Altman et al.	[4]	Demonstrates that system-wide ERAS implementation can standardise surgical care, reduce length of stay, and improve patient experience without increasing readmissions.
Pujić et al.	[5]	Shows that ERAS protocols for caesarean section are safe and lead to earlier mobilisation, faster recovery, and shorter hospital stay.
Cavallaro and Bordeianou	[6]	Describes practical steps for implementing an ERAS pathway in colorectal surgery, emphasising multidisciplinary collaboration and continuous audit.
Asklid et al.	[7]	Finds that robotic and laparoscopic rectal cancer surgery within an ERAS protocol results in better short-term outcomes compared with open surgery.
Grant and Engelman	[8]	Summarises overarching themes of ERAS Society guidelines, highlighting multimodal, evidence-based perioperative care and continuous quality improvement.
Wang et al.	[9]	Identifies organisational, cultural, and knowledge-based barriers to ERAS implementation in China, stressing the importance of multidisciplinary engagement.
Stavropoulou et al.	[10]	Explores nurses’ perceptions of quality of care, emphasising patient-centeredness, safety, teamwork, and adequate resources.
Kristensen et al.	[11]	Highlights the role of patient safety policies and contextual factors in sustaining perioperative safety improvements across European healthcare systems.
Shestopalova and Gololobova	[12]	Demonstrates that standard operating procedures improve healthcare safety by reducing variability and preventing errors.
Singh et al.	[13]	Shows that structured healthcare management protocols are associated with improved quality of patient care and organisational efficiency.
Mistri et al.	[14]	Reports that strengthening patient safety culture improves communication, reporting, and overall quality of care in hospitals.
Shaw et al.	[15]	Finds that certification and accreditation are associated with stronger quality management systems across multiple clinical services.
Ellis et al.	[16]	Describes successful ERAS implementation within an anaesthesia department, emphasising leadership, education, and protocol standardisation.
Taberna et al.	[17]	Demonstrates that a multidisciplinary team approach improves coordination of care and overall quality outcomes in oncology.
Pagano et al.	[18]	Presents a protocol for implementing consensus-based perioperative care pathways to reduce clinical variation in elective surgery.
Sattler et al.	[19]	Systematic review showing that enhanced recovery pathways in hip and knee arthroplasty reduce length of stay and improve early outcomes.
Tan et al.	[20]	Shows that ERAS implementation for hip replacement improves the quality of recovery in an Australian private hospital setting.
Jain et al.	[21]	Reviews recent advances and strategies in ERAS, highlighting education, protocol adherence, and audit as key success factors.
Wainwright	[22]	Discusses the “knowing–doing gap” in ERAS and emphasises the role of nurses and allied health professionals in quality improvement.
Balfour et al.	[23]	Identifies barriers and solutions for nurses implementing ERAS, including education, staffing, and organisational support.
Zhang et al.	[24]	Demonstrates that targeted nursing interventions within ERAS improve recovery and outcomes after minimally invasive spine surgery.

Quality of Care and Patient Safety

Quality of care refers to the extent to which healthcare services increase the likelihood of desired health outcomes and are consistent with current professional knowledge. Patient safety constitutes a fundamental dimension of quality and focuses on the prevention of errors, adverse events, and harm associated with healthcare delivery [10].

The perioperative environment is particularly susceptible to safety risks due to the complexity of procedures, time pressure, and the need for effective coordination among healthcare professionals. The implementation of standardised protocols and clinical pathways has been widely recognised as a key strategy for enhancing patient safety and improving the quality of care [11].

Within this framework, ERAS protocols align with contemporary quality and safety principles by providing structured guidance for perioperative management and promoting adherence to evidence-based practices.

Role of Protocols in Healthcare Quality and Safety Systems

Clinical protocols, standard operating procedures, and work instructions constitute core elements of modern healthcare quality management systems. Their primary purpose is to clearly define processes, ensuring consistency, reproducibility, traceability, and safety in care delivery [12].

Protocols support quality improvement by enabling systematic monitoring, evaluation, and continuous optimisation of healthcare processes. Through standardisation, unwarranted variability in clinical practice is reduced, and decision-making becomes more transparent and evidence-driven [13].

From a patient safety perspective, protocols function as preventive mechanisms that introduce safeguards within clinical workflows. By establishing clear steps and responsibilities, they reduce the likelihood of omissions and errors, while fostering a culture of patient safety among healthcare professionals [14].

Quality assurance frameworks, such as ISO 9001:2015, emphasise the importance of documented processes, risk-based thinking, and continuous evaluation of organisational performance. In parallel, accreditation programmes in surgical and oncological care, such as the Organisation of European Cancer Institutes (OECI), rely heavily on structured clinical pathways and multidisciplinary protocols. In this context, ERAS protocols can be regarded as functional clinical tools that integrate quality management principles into everyday perioperative practice [15].

ERAS Protocols: Core Principles

ERAS protocols are based on a series of evidence-based interventions applied throughout the perioperative continuum. Core principles include preoperative patient education, optimisation of nutritional status, minimisation of preoperative fasting, multimodal analgesia, early mobilisation, and early resumption of oral intake [8,16].

These interventions collectively aim to attenuate the perioperative stress response prior to surgery, preserve functional capacity, and enhance recovery. Importantly, ERAS protocols are designed to be adaptable, allowing for individualisation based on patient characteristics and surgical complexity, while maintaining adherence to core evidence-based elements [8].

The systematic application of ERAS principles has been associated with improved patient experiences and enhanced efficiency of perioperative care across various surgical disciplines.

ERAS Protocols and Improvement of Perioperative Care Quality

The adoption of ERAS protocols contributes to improved quality of perioperative care by promoting standardisation and reducing unwarranted variability in clinical practices. Clearly defined pathways facilitate a multidisciplinary approach and enhance continuity of care across the perioperative phase [13,17].

Furthermore, ERAS pathways actively involve patients in their recovery process through structured education and shared decision-making. Patient engagement has been linked to improved adherence to care plans and increased satisfaction with healthcare services.

From an organisational perspective, ERAS implementation supports quality monitoring and benchmarking, enabling healthcare institutions to evaluate performance and identify areas for improvement [11,18,19].

ERAS Protocols and Patient Safety

Patient safety represents a central objective of ERAS implementation. By integrating evidence-based practices, ERAS protocols reduce the risk of perioperative complications and adverse events. Early mobilisation, effective pain management, and avoidance of unnecessary invasive interventions contribute to a safer postoperative recovery [20].

Continuous monitoring of protocol adherence and outcomes reinforces a safety-oriented culture within perioperative multidisciplinary teams. ERAS pathways thus serve as a structured mechanism for translating patient safety principles into daily clinical practice [21].

Role of Peri-Anaesthesia Nursing Care

Nursing care plays a pivotal role in the successful implementation of ERAS protocols. In the PACU and immediate postoperative phase, peri-anaesthesia nurses are central to ERAS adherence through pain and symptom management (including nausea and vomiting), monitoring for early complications, facilitating early mobilisation when appropriate, and supporting discharge readiness.

Nurses are actively involved in patient education, coordination of care, and monitoring of compliance with protocol elements throughout the perioperative period [22].

Due to their continuous presence at the patient’s bedside, nurses are well-positioned to detect early signs of complications and to ensure timely interventions. Their contribution is essential for maintaining quality and safety standards, and for supporting interdisciplinary collaboration within ERAS programmes [23].

Strengthening the nursing role is, therefore, a prerequisite for sustainable ERAS implementation and for achieving optimal perioperative outcomes [24].

Discussion

The findings of this narrative review indicate that ERAS protocols play a significant role in improving the quality and safety of perioperative care across a wide range of surgical specialities. The reviewed literature consistently suggests that the structured and evidence-based nature of ERAS pathways contributes to reduced variability in clinical practice, improved coordination among healthcare professionals, and enhanced patient outcomes [8].

One of the key elements emerging from the reviewed studies is the contribution of ERAS protocols to patient safety. By incorporating standardised perioperative interventions, such as early mobilisation, optimised pain management, and early nutritional support, ERAS pathways address common risk factors associated with postoperative complications [16]. These findings align with international evidence, highlighting the importance of protocol-driven care in reducing adverse events and promoting safer surgical recovery.

Furthermore, ERAS protocols appear to support quality improvement by fostering multidisciplinary collaboration and continuity of care throughout the perioperative period. The integration of clearly defined roles and responsibilities within ERAS pathways enhances communication among surgical teams, anaesthesiologists, and nursing staff. This coordinated approach is particularly relevant in complex perioperative environments, where fragmentation of care may compromise quality and safety [13,17,20].

The role of nursing care emerged as a central factor in successful ERAS implementation. Nurses are actively involved in patient education, monitoring adherence to protocol components, and the early identification of complications. The reviewed studies highlight that effective nursing engagement not only facilitates protocol compliance but also strengthens patient participation in recovery, which is a recognised determinant of quality care [10,22,23].

These benefits are particularly relevant to peri-anaesthesia nursing practice, as many ERAS elements are operationalised during immediate recovery and in PACU care.

Despite the positive findings, several challenges related to ERAS implementation were identified. Organisational barriers, limited training, and resistance to change among healthcare professionals may hinder widespread adoption, particularly within healthcare systems facing resource constraints [9,23]. These challenges underscore the need for leadership support, continuous education, and the development of a culture that prioritises quality and patient safety. 

Within the Greek healthcare system, these challenges may be further exacerbated by resource limitations and variability in institutional support. Successful implementation, therefore, depends on strong leadership commitment, structured education programmes, and the cultivation of an organisational culture that prioritises quality improvement and patient safety. Addressing these factors is essential for the effective integration of ERAS protocols into routine perioperative practice.

This review has certain limitations. As a narrative review, it does not follow a systematic methodology, and the findings are based on a limited number of publications. Additionally, heterogeneity in study designs and outcome measures restricts direct comparison between studies. Nevertheless, the consistency of the reported benefits across different settings strengthens the overall conclusions.

## Conclusions

ERAS protocols constitute a comprehensive and evidence-based approach to enhancing the quality and safety of perioperative care across a wide spectrum of surgical specialities. Their systematic implementation supports the standardisation of clinical practice, reduces perioperative risk, and strengthens multidisciplinary teamwork, ultimately contributing to improved surgical patient outcomes.

Peri-anaesthesia nurses play a pivotal role in translating ERAS principles into practice during PACU care and the immediate postoperative period, directly influencing recovery quality and patient safety. Sustained ERAS implementation depends on effective nursing engagement, teamwork, and organisational support. Further research examining the application, adaptation, and evaluation of ERAS protocols within the Greek healthcare system is warranted to strengthen the evidence base and support continuous quality and safety improvement in healthcare services.
